# Grp94-specific monoclonal antibody to counteract BRAF inhibitor resistance in BRAF^V600E^ melanoma

**DOI:** 10.1186/1479-5876-13-S1-K12

**Published:** 2015-01-15

**Authors:** F Sabbatino, E Favoino, Y Wang, X Wang, V Villani, L Cai, L Yang, S Ferrone, CR Ferrone

**Affiliations:** 1 Department of Surgery, Massachusetts General Hospital, Harvard Medical School, Boston, Massachusetts, USA

## 

The development of BRAF-I resistance in BRAF^V600E^ melanoma underlies the need to develop strategies to counteract this resistance. It has been shown that administration of heat shock protein 90 (HSP90) inhibitors can counteract multiple mechanisms which drive BRAF-I resistance by reactivation of MAPK and activation of PI3K/AKT pathway. However the clinical application of this strategy is hampered the high toxicity associated with administration of currently available HSP90 inhibitors. To overcome this limitation we have developed a novel monoclonal antibody (mAb), named W9, which recognizes an extracellular epitope of glucose regulated protein of 94 kDa (Grp94), a member of the HSP90 family. The mAb W9 defined Grp94-epitope is selectively expressed on malignant cells but is not detectable on normal cells. Therefore targeting Grp94-epitope by mAb W9 is expected to cause limited if any side effects. The mAb W9 was found to increase and restore the sensitivity to BRAF-I of BRAF^V600E^ melanoma cells including cells which have acquired BRAF-I resistance because of PDGFRα upregulation.

